# Supplementary material for: Anti-Tumor Functions of Prelatent Antithrombin on Glioblastoma Multiforme Cells
Source: Biomedicines. 2021 May 7;9(5):523. doi: 10.3390/biomedicines9050523 (PMC8151964; doi:10.3390/biomedicines9050523)
Supplement: Supplementary file 1 [file biomedicines-09-00523-s001.zip › biomedicines-1142158-supplementary .pdf]

biomedicines

Supplementary Materials

Anti-Tumor Functions of Prelatent Antithrombin on Glioblastoma Multiforme Cells

Citation: Peñas-Martínez, J.; Luengo-Gil, G.; Espín, S.; Bohdan, N.; Ortega-Sabater, C.; Ródenas, M.C.; Zaragoza-Huesca, D.; López-Andreo, M.J.; Plasencia, C.; Vicente, V.; et al. Anti-Tumor Functions of Prelatent Antithrombin on Glioblastoma Multiforme Cells. *Biomedicines* **2021**, *9*, 523. https://doi.org/10.3390/biomedicines9050523

Academic Editor: Elizabeth Shinmay Yeh

Received: 25 February 2021
Accepted: 4 May 2021
Published: 10 May 2021

Publisher's Note: MDPI stays neutral with regard to jurisdictional claims in published maps and institutional affiliations.

Copyright: © 2021 by the author. Licensee MDPI, Basel, Switzerland. This article is an open access article distributed under the terms and conditions of the Creative Commons Attribution (CC BY) license (http://creativecommons.org/licenses/by/4.0/).

1. Prelatent Antithrombin Characterization

1.1. Electrophoretic Mobility and Structural Stability

8% Native and urea PAGE under non-reducing conditions analysis of AT conformations (0.17 μM) were performed using a discontinuous assay to evaluate electrophoretic mobility and structural stability. Antithrombin was detected by Western blot following standard immunoblotting procedures using polyvinylidene difluoride membrane, a rabbit anti-human antithrombin antibody (1:5000 dilution) (Sigma-Aldrich, Madrid, Spain) and a horseradish peroxidase-coupled donkey anti-rabbit IgG antibody (1:10,000 dilution) (NA 9340v, GE Healthcare, Barcelona, Spain). Antithrombin bands were visualized by the ECL kit detection (GE Healthcare, Barcelona, Spain) (Figure S1).

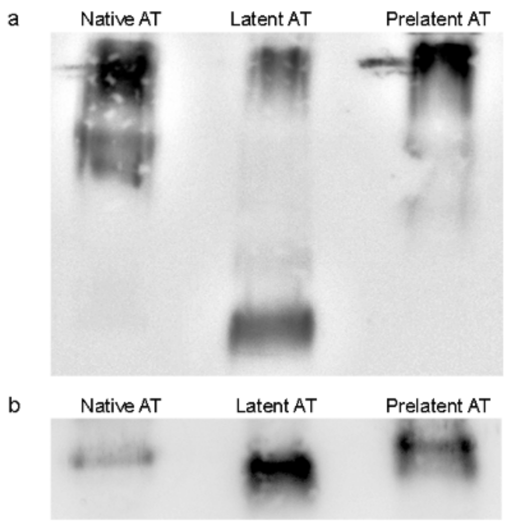

Figure S1. Electrophoretic mobility and structural stability of antithrombin conformations. Native (**a**) and urea (**b**) PAGE of native, latent and prelatent AT. Western blot was carried out with an anti-antithrombin antibody.

1.2. Heparin-Antithrombin Binding Assay. Dissociation Constant (K_D) Determination

The dissociation constant (K_D) for the interaction between antithrombin-heparin was determined as described previously [1]. Briefly, the increase of intrinsic fluorescence of native and prelatent antithrombin (50 nM) after pentasaccharide Fondaparinux (0–8 μM) titrations (Arixtra, Madrid, Spain) at room temperature (25 °C) under physiologic ionic strength (I) of 0.30 in 20 mM sodium phosphate, 0.25 M NaCl, 0.1 mM ethylenediaminetetraacetic acid, polyethylene glycol 8000 0.1%, pH 7.4, in a 2 ml quartz cuvette (10 mm path length) (Agilent technologies, Germany) was monitored on a Cary Eclipse Fluorescence Spectrophotometer (Agilent, Barcelona, Spain), with excitation at 280 nm and emission at 340 nm, using bandwidths of 3.5 nM for both excitation and emission. Fluorescence emission intensity was calculated as the average of 100 measurements saved at 1-s intervals for each heparin addition. Data were analyzed using SigmaPlot 11.0 software (Systat Software Inc., San Jose, CA, USA; accessed on 5 May 2018) and adjusting the results by non-linear regression to a 2-paremeter hyperbolic equation to estimate the K_D for antithrombin-heparin interactions (Figure S2).

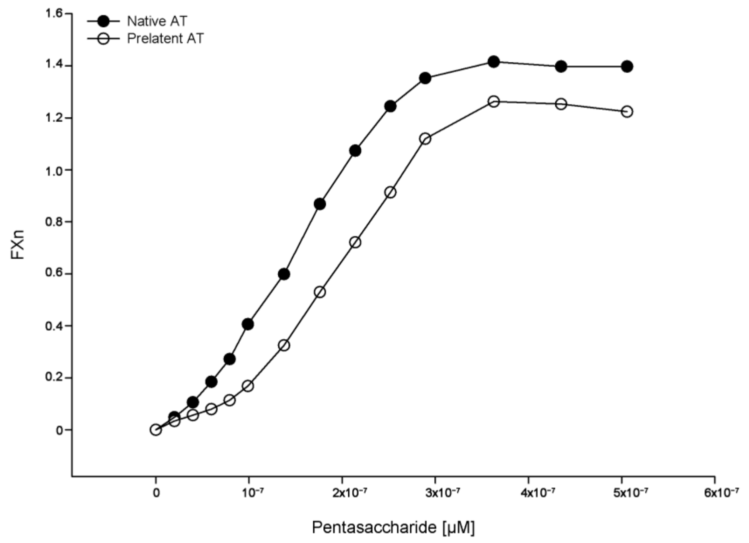

Figure S2. Heparin-antithrombin binding assay. Dissociation constant (K_D) determination. Plot of intrinsic tryptophan fluorescence intensity of native and prelatent antithrombin versus increasing heparin concentration. Black and white circles represent the best-fit curves calculated with non-linear regression to a 2-paremeter hyperbolic equation.

1.3. FXa and FIIa (Thrombin) Inhibition In Vitro

To characterize the interaction of the proteases (FXa or FIIa) and native or prelatent antithrombin, in absence or with increasing concentrations of Fondaparinux pentasaccharide (0–2 nM) for FXa or unfractionated heparin (0–1.2 nM Heparin Hospira 1%) for FIIa, reactions were started by addition of chromogenic substrate S2238 for FIIa or S2765 for FXa (200 µM) (Instrumentation Laboratory Company, Milan, Italy) to a mixture containing the proteases and inhibitor (prelatent or native antithrombin, 0.05 µM) pre-incubated for 2 minutes at room temperature. All reagents were diluted in the reaction buffer 20 mM sodium phosphate, 100 nM NaCl, 0.1 mM ethylenediaminetetraacetic acid, 0.1% polyethylene glycol 8000, at pH 7.4. Reactions were performed in 96-well flat-bottom plates at 37 °C in a total volume of 200 µL. The enzymatic residual activity was determined using a Synergy HT microplate reader and Gen 5 1.11 software, measuring absorbance at 405 nm. Results were analyzed and represented using SigmaPlot 11.0 software (Table S1).

Table S1. Rate constants for FXa and FIIa (thrombin) inhibition in vitro.

Condition	FXa	FXa + Heparin	FIIa	FIIa + Heparin
Native antithrombin	9.40×10^4	1.67×10^8	4.80×10^4	4.17×10^7
Prelatent antithrombin	7.20×10^4	1.67×10^8	5.80×10^4	7.50×10^6

Native and prelatent antithrombin were compared with respect to the rate constant (k) (M^{-1} s^{-1}) of inhibition of FXa and FIIa (thrombin), in the absence or presence of heparin (pentasaccharide or unfractionated heparin, respectively), and reaction kinetic curves were calculated as a function of time or heparin concentration. Represented results are the mean of 3 independent experiments.

Formation of covalent complexes between native or prelatent antithrombin and FXa or FIIa was evaluated by incubating for 15 minutes at 37 °C the antithrombin conformations with Fondaparinux pentasaccharide (0.14 mM) or unfractionated heparin (3.3 mM) and, next and respectively, with FXa (1 µM) or FIIa (9.50 µM) for 45 minutes at 37 °C. The same assay was carried out but in the absence of heparin. Complexes were evaluated by 8% SDS-PAGE under non-reducing conditions and silver staining (Figure S3).

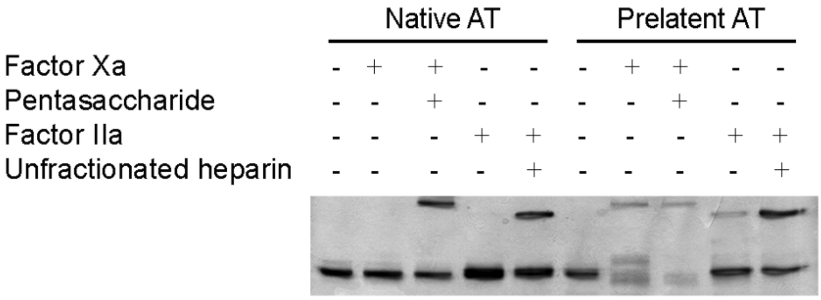

Figure S3. Formation of complex of antithrombin with FXa and FIIa, in the presence or absence of heparin. SDS-PAGE under non-reducing (-DTT) conditions of native and prelatent antithrombin in complex with FXa or FIIa, in the presence or absence of heparin (pentasaccharide or unfractionated heparin, respectively), and silver staining.

2. Effect of Dose Dependent Concentration of Prelatent Antithrombin on Cell Migration

To evaluate prelatent AT effect on U-87 MG cells migration, we first determined by wound healing assay the concentration at which the prelatentantithrombin exerts the greatest inhibitory effect on this process. U-87 MG cells were cultured as confluent monolayers in a polystyrene microplate 6-well (ThermoFisher Scientific, Madrid, Spain). Then, cells were wounded by removing a 300–500 μm-wide strip of cells across the well with a standard 200 μl pipette tip, and washed twice to remove non-adherent cells. Increasing concentrations of prelatent antithrombin were incubated with cells for 16h: (1) Control: 0 μM, (2) 0.86 μM, (3) 1.72 μM, (4) 2.16 μM, (5) 2.59 μM, (6) 3.02 μM. Finally, woundhealing was quantified using ImageJ software as the median percentage of the remaining cell-free area comparedto the area of the initial wound. Triplicate assays were performed for each concentration (Figure S4).

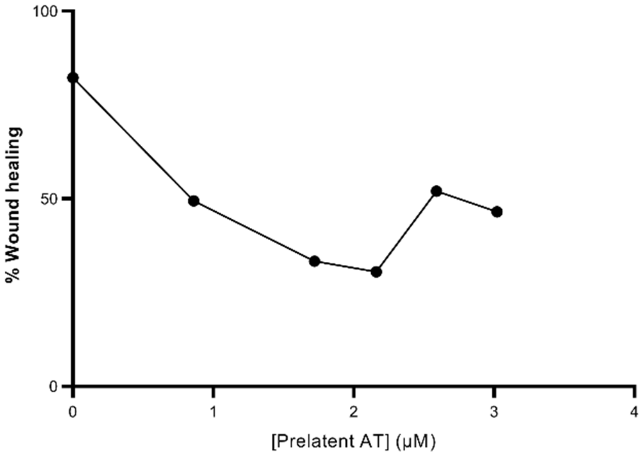

Figure S4. Dose-response curve of prelatent antithrombin and percentage of wound healing. Cells were treated with different concentrations of prelatent AT for 16 hours. The results are expressed as the percentage of remaining wound area. All values are represented as the means ± S.D.

3. Effect of Prelatent Antithrombin on Proliferation of Glioblastoma Cells

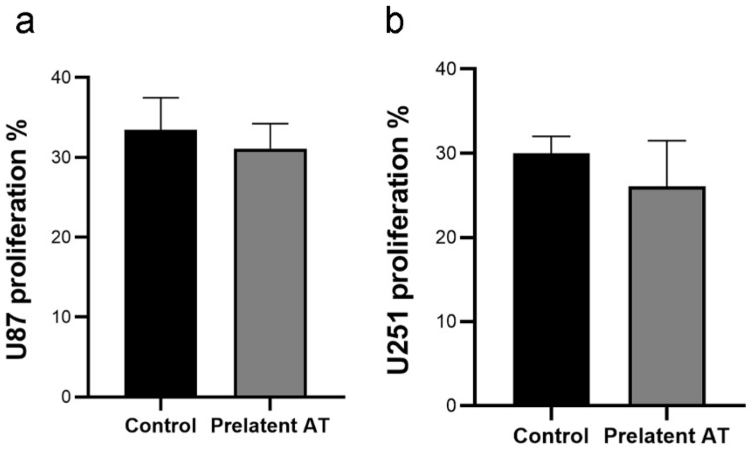

Figure S5. Effect of prelatent AT on the proliferation of human GB cells measured by the BrdU incorporation assay. (**a**) U-87 MG and (**b**) U-251 MG cells treated with buffer A (control) and prelatent AT (2.16 μM). Each bar indicates the mean ± SD, *n* = 3.

4. Effect of Prelatent Antithrombin on Epithelial-Mesenchymal Transition Gene Expression

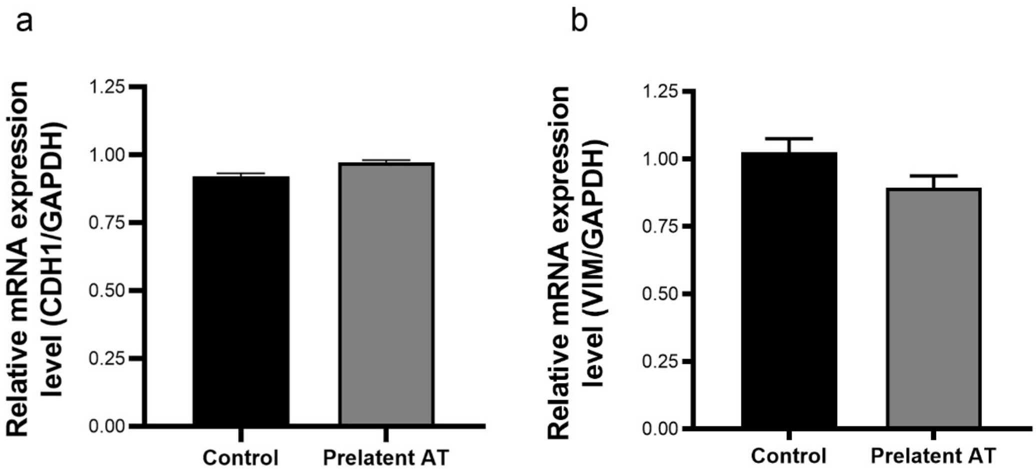

Figure S6. Relative expression of *CDH1* (**a**) and *VIM* (**b**) to GAPDH mRNA (2-ΔCt). Each condition was evaluated in triplicate, and 5 different samples per group were analyzed.

5. Effect of Prelatent Antithrombin on the Expression of Different Cancer Signaling Molecules in U-251 MG Cells

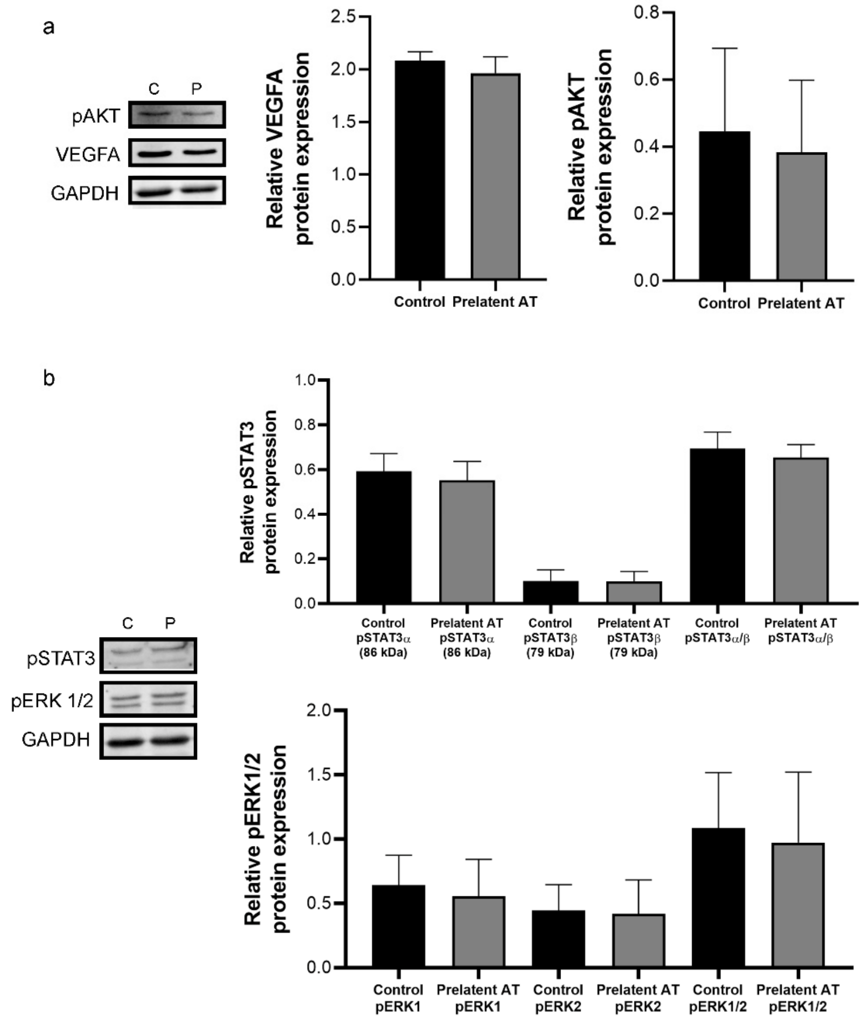

Figure S7. VEGFA, pSTAT3, pAKT, and pERK1/2 expression on U-251 MG cells. (**a**) Electrophoresis and western blot of VEGFA and pAKT in lysates of U-251 MG cells treated with buffer (C) or prelatent antithrombin (P). GAPDH expression was detected as loading control. (**b**) Electrophoresis and western blot of pSTAT3 and pERK1/2 in lysates of U-251 MG cells treated with buffer (C) or prelatent antithrombin (P). GAPDH expression was detected as loading control. AT: antithrombin.

References

1. Langdown, J.; Belzar, K.J.; Savory, W.J.; Baglin, T.P.; Huntington, J.A. The Critical Role of Hinge-Region Expulsion in the Induced-Fit Heparin Binding Mechanism of Antithrombin. *J. Mol. Biol.* **2009**, *386*, 1278–1289, doi:10.1016/j.jmb.2009.01.028.
